# Benzodiazepine use disorder: A cross-sectional study at a tertiary care center in Lebanon

**DOI:** 10.1097/MD.0000000000030762

**Published:** 2022-09-23

**Authors:** Tharwat El Zahran, Elie Kanaan, Lynn Kobeissi, Joseph Bouassi, Aseel Sarieddine, Joseph Carpenter, Ziad Kazzi, Eveline Hitti

**Affiliations:** a Department of Emergency Medicine, American University of Beirut Medical Center, Beirut, Lebanon; b Department of Emergency Medicine, Emory University, Atlanta, GA, USA.

**Keywords:** abuse and dependence, benzodiazepine, drug misuse, substance use disorder

## Abstract

Benzodiazepines are medications used for the treatment of multiple conditions including anxiety disorders, insomnia, agitation, and seizures. They are the most prescribed psychiatric medications and the third most misused drugs among adults and adolescents in the US. This study aims to assess the patient utilization patterns and benzodiazepine use disorder among Lebanese patients. A cross-sectional study was performed on Lebanese patients presenting to the Emergency Department of the American University of Beirut Medical Center (AUBMC), between November 11th, 2019, and May 30th, 2020. Institutional review board approved the study, and an informed consent was obtained from patients. A total of 244 patients were included in the final analysis. A total of 154 (63.1%) patients were found to have benzodiazepine use disorder as per the DSM-V criteria with the majority (64%) being females and young adults aged 18 to 40 years. The most common medication was alprazolam, and anxiety was the most common reason for benzodiazepine use. The majority (88%) of patients obtained their medications using a physician’s prescription. More than half of users were not satisfied with the physician’s instructions and lacked knowledge about side effects and abuse potential. The high rate of benzodiazepine misuse among our young adults highlights an important public health concern that requires interventions and policy implementation.

## 1. Introduction

Benzodiazepines are medications used for the treatment of multiple conditions including anxiety disorders, insomnia, agitation, seizures, and induction of amnesia.^[1]^ They are the most prescribed psychiatric medications and the third most misused drugs among adults and adolescents in the US.^[2]^ Despite their efficacy, benzodiazepines are associated with an addictive potential and, if used inappropriately can lead to significant morbidity and mortality.

Benzodiazepines became increasingly prescribed in the late 1990s and early 2000s. According to Bachhuber et al,^[3]^ benzodiazepine prescribing in the United States increased by 67% from the mid-1990s to 2013, with a 3-fold increase in the number of benzodiazepines prescribed. This rise has been accompanied by accumulating evidence of the toxicity and addictive potential of benzodiazepines. According to Maust et al,^[4]^ approximately 2.2% of the United States (US) population misused benzodiazepines in 2015 and 2016. Data from the National Institute on Drug Abuse^[5]^ revealed that the number of benzodiazepine-related deaths has steadily increased from less than 1000 in 1999 to nearly 9000 in 2015. The use and misuse of benzodiazepines have also contributed significantly to the current opioid overdose epidemic. It is reported that benzodiazepines were involved in ~30% of opioid overdose deaths in 2015 (National Institute on Drug Abuse, 2018). New concerns have recently emerged due to the increasing availability of highly lethal benzodiazepines on the illicit market (e.g., illicitly produced pills that combine benzodiazepines with fentanyl and potent “designer” benzodiazepines).^[6]^

The reasons for the increased use and misuse of benzodiazepines are multifaceted and are attracting increasing attention as fears of a benzodiazepine epidemic rise in different parts of the world. In addition to the pattern of increased prescribing of benzodiazepines by physicians, Lembke et al^[7]^ showed that patients are not aware of the addiction potential is another factor. Additionally, patients who have developed a dependence on benzodiazepines do not get adequate support and guidance to help them discontinue their use of these medications.^[8]^

Benzodiazepine use and misuse are particularly problematic in many middle eastern countries where access to benzodiazepine is not controlled, but rather easily available at pharmacies without the need for prescriptions (Khalife et al). In Pakistan, benzodiazepine overdoses represented 84% of self-poisoning cases.^[9]^ In Jordan, benzodiazepines were also frequently cited as a drug of abuse.^[10]^ After excluding nicotine, benzodiazepines were the most abused substance among Egyptian adolescents.^[11]^ In Lebanon, a study by Hitti et al^[12]^ showed that benzodiazepine overdoses accounted for the most common pharmaceutical drugs of overdose along with sedatives, hypnotics, and antipsychotics.

In Lebanon, the literature on benzodiazepine uses and misuse is scarce. In 2000, Naja et al explored the prevalence of individuals who have used benzodiazepines within the last month in a sample of 1000 random participants. The authors noted that 9.6% of respondents had used benzodiazepines in the prior month. Additionally, they estimated that the dependence rate in 400 benzodiazepine users was ~50.2%.^[13]^ In 2016, Ramadan et al^[14]^ studied randomly selected Lebanese benzodiazepine users that were a sampled from 27 different pharmacies allocated across different regions of Lebanon and noted that a large proportion was using benzodiazepine as a long-term treatment for anxiety although it is recommended only for short-term use.

This study aims to evaluate the patient utilization patterns of benzodiazepines use and benzodiazepine use disorder among emergency medicine (ED) visit patients in the largest academic medical center ED in Lebanon.

## 2. Material and methods

### 2.1. Study design and setting

This study is a cross-sectional survey that assesses benzodiazepine use and benzodiazepine use disorder (BUD) among Lebanese patients presenting to the emergency department of the American University of Beirut Medical Center (AUBMC), between November 11th, 2019, and May 30, 2020. The AUBMC is the largest tertiary care center in Lebanon. The center has 358 beds and receives approximately 55,000 ED visits and approximately 25,000 inpatient admissions annually. Adult patients comprise 80% of the ED visits and 83% of hospital admissions. Most ED patients (75%) are covered through private insurance, whereas 23% pay out of pocket, and 2% are covered through governmental insurance. The study protocol was approved by the Institutional Review board at the American University of Beirut Medical Center (study protocol SBS 2019-0304). A written informed consent was obtained from patients. This study conforms with the Strengthening the reporting of observational studies in epidemiology guidelines and a complete checklist has been uploaded as “Supplemental Digital Content (supplemental table, http://links.lww.com/MD/H388)”.

### 2.2. Selection of participants

Patients presenting to the ED who report using benzodiazepines as part of their home medications were identified through the electronic health system (Epic Systems, Verona, WI). The research assistants received an electronic message through EPIC 24/7, to alert them of potential subjects. Some participants were initially recruited during their ED visit and some were recruited 2 months later due to the unexpected restriction of ED visits when local cases of coronavirus disease 2019 (COVID-19) infections increased, and hospitalizations surged in Lebanon during the study period.

**Figure 1:**
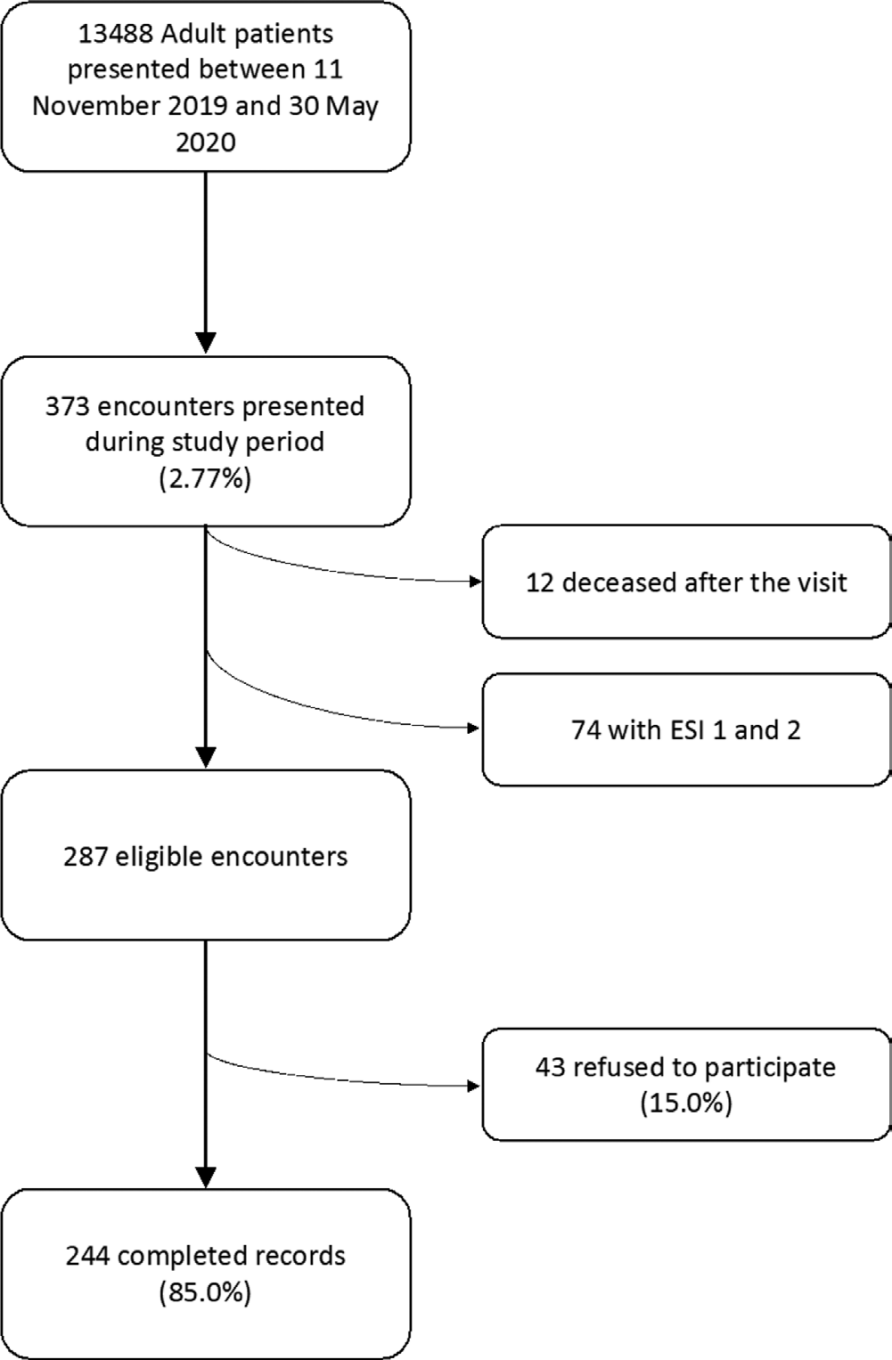
Flowchart of included and excluded patients.

All adult patients (>18 years of age) who reported taking benzodiazepines were included in the study. Patients were excluded if they do not speak English or Arabic and those with an emergency severity score of 1 or 2 (i.e., their state was deemed unstable and for recruitment into the study). Participants who refused to participate and those who had died during period from initial ED visit and time of contact were excluded (Fig. 1). Written informed consent was obtained from the patients who were enrolled before the COVID-19 infection surge. The remaining patients were contacted by phone and verbal consent was obtained with a phone-based questionnaire.

### 2.3. Study measures

To assess BUD, a validated questionnaire based on the national survey on drug use and health and the diagnostic and statistical manual of mental disorders V (DSM-V) was used. The questionnaire was administered in Arabic or English “Supplemental Digital Content (Appendix A, http://links.lww.com/MD/H387)”.

Demographic data were collected, including age, gender, marital status, educational level, and employment status. Information on the patient’s health was also collected and included perceived health status, chronic medical problems, current medications, presence of psychiatric comorbidities, and primary care physician/psychiatrist follow-up. Patients were also asked about their history of substance use. Additionally, all detailed information on benzodiazepine uses in the last 6 months, including frequency, duration, medication use, the reason for its use, co-use with other nonbenzodiazepine anxiolytics, and ways to obtain the medication were collected. Information on perceptions of use, misuse, and dependence was also collected.

### 2.4. Statistical analysis

The Statistical Package for Social Sciences (IBM SPSS 25.0) was employed to execute the statistical analysis. Frequency and percentage tables represented Categorical variables whilst means and standard deviations (mean ± SD) were used for continuous variables. The Chi test was applied since most of the variables were categorical to observe the associations between the different demographic, socioeconomic, exposure variables and our outcome. To test for any associations between our outcome, BUD, and other continuous variables we used Student *t* test and Fisher exact test depending on the samples. To control for confounding variables, bivariate analysis was utilized. A *P* value of <.05 was considered statistically significant.

## 3. Results

Between November 11th, 2019, and May 30th, 2020, 13, 488 patients older than 18 years of age presented to the ED at AUBMC, of which 373 (2.77%) reported taking benzodiazepines. All patients on benzodiazepines were contacted; 12 were deceased by the time of contact, and 74 patients were excluded because of ESI 1 and 2. Eligible patients were 287. Among those, 43 (15%) refused to participate so 244 (85%) were included in the final study. Most patients (194) were contacted through phone after their ED visit due to COVID-19 restrictions to the ED. The rest (50) of the patients were recruited during their ED visit.

Among the included patients (244), a total of 154 (63.1%) patients were found to have BUD as per the DSM-V criteria.

### 3.1. Demographics and general health

Of all the 244 participants, 155 (63.5%) were females and the mean age was 55.8 ± 16.9 years with a range of 18 to 96 years. Most patients were aged between 41 and 68 years (53.3%), while 19.3% were between 18 and 40 years of age and 27.5% were 66 years or older. Most of the participants were married (69.3%), had a university-level education (59.0%), and were not employed at the time of recruitment (60.2%) (Table 1).

**Table 1:** The characteristics of benzodiazepine users presenting to the emergency department at the American University of Beirut Medical Center.

Variable	Count (%)	Benzo use disorder N (%)		*P* value
		Yes	No	
		154 (63.1)	90 (36.9)	
Mean age (yr)		53.5 ± 15.9	59.7 ± 18.1	.006
Age groups				.006
18–40	47 (19.3)	35 (74.5)	12 (25.5)	
41–68	130 (53.3)	87 (66.9)	43 (33.1)	
≥66	67 (27.5)	32 (47.8)	35 (52.2)	
Gender				.011
Female	155 (63.5)	107 (69.0)	48 (31.0)	
Male	89 (36.5)	47 (52.8)	42 (47.2)	
Marital status				.849
Married	169 (69.3)	106 (62.7)	63 (37.3)	
Not married	75 (30.7)	48 (64.0)	27 (36.0)	
Level of education				.611
Less than university level	100 (41.0)	65 (65.0)	35 (35.0)	
University level	144 (59.0)	89 (61.8)	55 (38.2)	
Employment status				.451
Currently employed	97 (39.8)	64 (66.0)	33 (34.0)	
Not currently employed	147 (60.2)	90 (61.2)	57 (38.8)	
Presence of psychiatric diseases (as perceived by the patient)				<.0005
Yes	135 (55.3)	100 (74.1)	35 (25.9)	
No	109 (44.7)	54 (49.5)	55 (50.5)	
History of substance use				<.0005
Yes	183 (75.0)	128 (69.9)	55 (30.1)	
No	61 (25.0)	26 (42.6)	35 (57.4)	
Tobacco smoking				.003
Yes	164 (67.2)	114 (69.5)	50 (30.5)	
No	80 (32.8)	40 (50.0)	40 (50.0)	
Alcohol				.045
Yes	73 (29.9)	53 (72.6)	20 (27.4)	
No	171 (10.1)	101 (59.1)	70 (40.9)	
Opioids*				.264
Yes	8 (3.3)	7 (87.5)	1 (12.5)	
No	236 (96.7)	147 (62.3)	89 (37.7)	
MDMA*				.532
Yes	2 (0.8)	2 (100)	0 (0.0)	
No	242 (99.2)	152 (62.8)	90 (37.2)	
Marijuana*				.492
Yes	9 (3.7)	7 (77.8)	2 (22.2)	
No	235 (96.3)	147 (62.6)	88 (37.4)	
Cocaine*				.028
Yes	8 (3.3)	8 (100)	0 (0.0)	
No	236 (96.7)	146 (61.9)	90 (38.1)	

MDMA = 3,4-methylenedioxymethamphetamine.

* Calculated via Fisher exact test.

Among all participants, (N = 154, 63.1%), younger patients (*P* = .006) and those of female gender (*P* = .01), were significantly more likely to have BUD (Table 1).

When asked about their perception of having any form of psychiatric disease, 135 (55.3%) reported that they believe they do have a psychiatric illness, and 116 (85.9%) of these reported that they had seen a psychiatrist (Table 1).

Most patients reported having chronic diseases such as hypertension, coronary artery disease, cancer, thyroid disease, benign prostatic hyperplasia, migraine, diabetes, pulmonary disease, arrhythmias, autoimmune diseases, epilepsy, dyslipidemia, kidney disease, fibromyalgia, and others. Most reported following up with other physicians for various diseases. Participants with a perceived psychiatric illness were 1.5 times more likely to have BUD (*P* < .0005). One hundred and fifteen (47.1%) participants perceived their health to be poor/fair, while 31.1 % reported it to be good, and 21.7 % perceived their health to be very good/excellent.

### 3.2. History of substance use

Most participants (75.0%) reported a history of substance use such as tobacco smoking (67.2%), alcohol (29.9%), opioids (3.3%), 3,4-Methylenedioxymethamphetamine (0.8%), marijuana (3.7%), and cocaine (3.3%) use. Moreover, 31 (12.7%) participants reported co-ingestion of Deanxit (flupentixol/melitracen), which contains a typical antipsychotic and a tricyclic antidepressant. Participants with any history of substance use were 1.6 times more likely to have BUD (*P* < .0005). Participants with a history of smoking tobacco (*P* = .003), alcohol use (*P* = .045), and cocaine use (*P* = .028) were, respectively, 1.4, 1.2, and 1.6 times more likely to have BUD (Table 1).

### 3.3. Benzodiazepine use

The most commonly used benzodiazepine medication was alprazolam (48.8%), followed by bromazepam (36.1%), clonazepam (18.0%), lorazepam (10.2%), diazepam (6.6%), and chlordiazepoxide (5.3%). There was no statistically significant association between the use of any benzodiazepine and having BUD.

Most participants (70.5%) have been using their benzodiazepines for more than 1 year, with 68 (27.9%) reporting that they have been using them for at least 10 years (Table 2). Participants who have been using their benzodiazepines for more than 1 year were 1.3 times more likely to have BUD (*P* = .030). Most participants reported that they take their benzodiazepines daily (74.2%).

**Table 2:** The patterns of benzodiazepine use among patients presenting to the emergency department at the American University of Beirut Medical Center.

Variable	Count (%)
Type of benzodiazepine	
Alprazolam	119 (48.8)
Bromazepam	88 (36.1)
Clonazepam	44 (18.0)
Lorazepam	25 (10.2)
Diazepam	16 (6.6)
Chlordiazepoxide	13 (5.3)
Duration of benzodiazepine use	
≤1 yr	72 (29.5)
Between 1 and 10 yr	104 (42.6)
≥10 yr	68 (27.9)
Frequency of benzodiazepine use	
Occasionally	63 (25.8)
Daily	181 (74.2)
Reason for benzodiazepine use	
Anxiety	144 (59.0)
Insomnia	111 (45.5)
Get high	61 (25.0)
Depression/low mood	57 (23.4)
Panic attacks	23 (9.4)
Spasticity	12 (4.9)
Seizures	6 (2.5)
Other*	17 (7.0)
How did you learn about benzodiazepines?	
From physician	213 (87.3)
From friends/family	25 (10.2)
On my own	9 (3.7)
How do you obtain the benzodiazepines?	
From the pharmacist, with prescription	215 (88.1)
From the pharmacist, without prescription	16 (6.6)
From friends/family	13 (5.3)

* Other: Alcohol withdrawal, cancer, tremor, fibromyalgia.

Anxiety was the most reported reason for benzodiazepine use (59.0%), followed by insomnia (45.5%), “wanting to get high” (25.0%), depression (23.4%), panic attacks (9.4%), spasticity (4.9%), and seizures (2.5%) (Table 2). Participants who take benzodiazepines for anxiety (*P* = .014) or insomnia (0.034) were respectively, 1.3 and 1.2 times more likely to have BUD.

Most patients learned about benzodiazepines from their physician (87.3%), while some (10.2%) reported that they learned about them from their friends/family or alone (3.7%). Most participants reported that they obtain their medication exclusively from the pharmacist with a physician’s prescription (88.1%) while the rest (11.9%) reported that they sometimes obtain it from friends/family members or without a prescription (Table 2). Participants who obtain their benzodiazepines from friends/family are 1.4 times more likely to have BUD (*P* = .006).

### 3.4. Awareness about benzodiazepine use

The vast majority of participants reported that they are confident about what their benzodiazepines are used for (78.1%), as well as how (74.8%) and when (76.0%) to use them. On the contrary, most participants reported that they were unable to name what other medications (71.9%), or foods/drinks (69.8%) cannot be consumed with their benzodiazepines. One hundred and ten participants (46.4%) reported that they were not satisfied with the overall explanation they received from their physician concerning their benzodiazepine medications. Approximately one-third (34.7%) of the participants reported that they were aware of the possible side effects of their benzodiazepines and only 43.4% were aware that there is a chance of becoming addicted to them (Table 3). Participants who reported that they were aware of the addictive potential of benzodiazepines were more likely to have BUD (*P* = .004).

**Table 3:** The awareness and knowledge of benzodiazepine users about their medication.

Statement (N = 242)	No	Neutral	Yes
Confident that they know what the benzodiazepines are for	24 (9.9%)	29 (12.0%)	189 (78.1%)
Can confidently describe how to use the benzodiazepines	32 (13.1%)	29 (12.0%)	181 (74.8%)
Can confidently describe when to use the benzodiazepines	33 (13.6%)	25 (10.3%)	194 (76%)
Can name the medications that cannot be taken with benzodiazepines	174 (71.9%)	25 (10.3%)	43 (17.8%)
Can name the foods/beverages that cannot be consumed with benzodiazepines	169 (69.8%)	18 (7.4%)	55 (22.7%)
Satisfied with the overall explanation received from the doctor concerning the benzodiazepines	110 (46.4%)	35 (14.8%)	92 (38.8%)
Understand the possible side effects of benzodiazepines	139 (57.4%)	19 (7.9%)	84 (34.7%)
Aware that there is a chance of becoming addicted to benzodiazepines	101 (41.7%)	36 (14.9%)	105 (43.4%)

## 4. Discussion

The use and misuse of benzodiazepines in Lebanon is an important public health concern that needs further characterization and mitigation strategies. In this cross-sectional study, we assessed the patient utilization patterns of benzodiazepines use and benzodiazepine use disorder (BUD) among ED patients presenting to the largest ED in Lebanon. Our findings show that more than half of the participants have BUD with the majority being females and young adults aged 18 to 40 years. Concomitant use of other substances of abuse, smoking, and alcohol was associated with higher BUD. Participants who used benzodiazepine for >1 year and who obtained them from friends and family were more likely to have BUD. Even though most patients obtained benzodiazepines using prescriptions, almost half of the users reported that they did not receive an adequate explanation from their physicians regarding benzodiazepine use. The majority of benzodiazepine users lacked knowledge regarding the side effects and addiction potential.

While 2 other studies^[13,14]^ assessed the prescribing patterns and prevalence of benzodiazepines among the Lebanese population, our study is the first to assess the Lebanese population with BUD presenting to the ED in the largest tertiary care center in Lebanon. Naja et al^[13]^ assessed the prevalence of benzodiazepine use and its associated sociodemographic risk factors using a national survey in 2000. They also assessed the dependence in 406 patients who reported at least 1 intake of benzodiazepine in the last month, using DSMIV criteria without assessing withdrawal. Ramadan et al^[14]^ assessed in 2016 the prescribing patterns among ambulating adult patients visiting pharmacies with BDZ prescriptions, without assessing misuse behavior. In their study, they found that the most commonly used benzodiazepine was alprazolam followed by bromazepam, which is similar to our findings. Additionally, they found that one-third of their patients reported experiencing side effects.

Interestingly, in our study, 63.1% of our benzodiazepine users were found to have a BUD. This is much higher than what is reported in many other countries. A study by Maust et al^[4]^ showed that benzodiazepine misuse accounted for ~20% of all adult US users in 2015 to 2016. A systematic review by Votaw et al^[2]^ assessed the epidemiology of benzodiazepine misuse in various countries. They found that in 2017, benzodiazepines and other tranquilizers were the third most misused illicit or prescription drug in the US. Available data from countries outside of the US such as Sweden, Thailand, Australia, and Spain, suggest that the rates of benzodiazepine misuse are relatively similar to those reported in the US.^[2]^ The increasing rates of benzodiazepine misuse could be related to over prescribing by physicians. The number and quantity of benzodiazepine prescriptions increased from the mid-1990s to 2013.^[2]^ Reports have shown that the use and misuse of benzodiazepines have contributed substantially to the current opioid overdose epidemic, with benzodiazepines involved in nearly 30% of opioid overdose deaths in 2015 (National Institute on Drug Abuse, 2018).^[2]^ In our study, 88% of our patients who used benzodiazepine obtained it from the pharmacy using a doctor’s prescription and 42% of our patients have used benzodiazepine for more than 1 year. A study was done by Ramadan et al^[14]^ in Lebanon over 2 years (2012–2014) showed that among all prescribers, cardiologists and psychiatrists tend to prescribe BDZs for a longer period. Our study results show that patients who have been using their benzodiazepines for more than 1 year were 1.3 times more likely to have BUD.

When assessing the benzodiazepine type, we found that almost half of our patients used alprazolam followed by bromazepam. This is similar to what was previously reported in Lebanon by Ramadan et al. Based on national ED visit data, alprazolam is the second most common prescription medication and the most common benzodiazepine to be involved in ED visits related to drug misuse (SAMHSA, 2013).^[15]^ CDC prescription death rate data reveal that between 2003 and 2009, alprazolam had the highest death rate increase of all benzodiazepines.^[15]^ In a study done by Hitti et al^[12]^ reporting on toxicological exposures among the Lebanese population, sedative/hypnotics/and antipsychotics accounted for the most common cause of pharmaceutical overdose with alprazolam topping the list. In our current study, we did not find any significance between the use of any specific benzodiazepine type and BUD. However, alprazolam has been shown to have a high misuse liability and is associated with severe withdrawal effects due to its unique pharmacokinetic properties of rapid absorption, low lipophilicity, high potency, and short half-life (*t*_1/2_).^[15]^

When assessing age and reasons for use among users with a BUD, younger patients, and those with anxiety were significantly more likely to have benzodiazepine use disorder. Interestingly, 74.5% of the patients with BUD were among the young adults aged 18 to 40 years, followed by the 41 to 68 age group. Our rates are comparable to the data from the 2015 to 2016 national survey on drug use and health, where the highest rates of combined sedative/tranquilizer misuse were also among 18 to 25-year-olds.^[2]^ Alarmingly, BUD prevalence is shifting over the past years from the older population to the younger adults. Ramadan et al^[14]^ assessed the profile of benzodiazepine use among 768 users in Lebanon during 2012 to 2014. In their study, 78.8% of users were aged 40 years and above versus 21.2% who were young adults [20–39] years of age. Similarly, Olfson et al^[16]^ reported that in 2008, patients who are older than 65 years of benzodiazepines users in the USA have recorded to have the highest rates of BUD, whereas in 2019, Maust et al,^[4]^ showed a decline in BUD percentage in this same age group and an increase of BUD in younger age groups; High rates of benzodiazepine misuse among adolescents and young adults are particularly concerning given that younger age of benzodiazepine misuse onset is associated with greater risk for and more rapid development of sedative, hypnotic, anxiolytic disorder.^[2]^ Anxiety and insomnia were the most common reasons for benzodiazepine use and were associated with a higher BUD. The increased rate of misuse among young adults may be related to increased anxiety and diminished job opportunities among the Lebanese young adults in the last few years due to political unrest and financial collapse.

Additionally, the majority (63%) of our benzodiazepine users were female. Females were more likely to have a BUD. This pattern of female predominance was similarly reported by Ramadan et al and Naja et al.^[13,14]^ Additionally, this pattern of female predominance was also prevalent in many other countries like the USA, Germany, and Switzerland where women had a higher predominance of BUD compared to men.^[16–19]^ Gender differences in benzodiazepine use can be explained in part, by the gender difference in psychiatric diseases. Asnaani et al^[17]^ reported that women are more likely to have social anxiety disorder compared to men. Another explanation could be due to gender bias among physicians’ prescribing patterns when assessing somatic complaints among females. A study by Clause et al on physician’s gender bias when assessing nonspecific, functional, and somatoform (NFS) syndromes found that females were more likely to be assessed as having an NFS syndrome compared to males with the same complaint. Additionally, when women’s pain is attributed to NFS, their rehabilitation could be prolonged as other alternative diagnoses and treatments were not sought and their satisfaction and treatment compliance is diminished.^[20]^

Additionally, participants with a history of smoking, alcohol use, or cocaine use had significantly higher rates of BUD. On the other hand, opioid co-ingestion was not associated with a statistically significant higher rate of BUD in contrary to what Jones et al^[21]^ found in their review of opioid and benzodiazepine combination use in the US, where 73% of heroin users were exposed to benzodiazepine use 1 year before entering rehabilitation. This low pattern of opioid co-exposure in Lebanon is likely due to stricter control on the prescription and availability of opioids in Lebanon compared to benzodiazepines. The use of narcotics and psychoactive drugs without a medical prescription is classified as a crime in Lebanon with an imprisonment sanction varying between 3 months and 3 years in addition to a financial fine.^[22]^

While the majority obtained their benzodiazepine using a doctor’s prescription, around half of the users reported that they were not satisfied with the overall explanation received from the doctor concerning the benzodiazepines. Additionally, 57% of the participants reported that they were not aware of the possible side effects of their benzodiazepines and only one-third of them were aware that there is a chance of becoming addicted to them. We did not explore the specialty of the prescribing physicians, and this could be a driver of over-prescribing if physicians with inadequate training/experience are the ones prescribing benzodiazepines in the Lebanese context. Furthermore, this highlights the inadequate patient-doctor communication regarding the safety and misuse potential of benzodiazepines. A systematic review by Mokhar A et al^[20]^ showed that patient information and educational strategies for HCPs can effectively lead to the appropriate use and prescription of benzodiazepines.

## 5. Conclusion

In conclusion, the high rate of benzodiazepine misuse among our population in young adults highlights an important public health concern that requires interventions and policy implementation. Even though a law banning benzodiazepine sales without a prescription from a doctor was passed in June 2001, the rates of misuse and abuse among the youth continue to rise. Young adults can still get benzodiazepine without prescription and in the black market. According to the national report from the National Observatory on Drugs and Drug Addiction,^[22]^ there is a 233% reported increase of several persons who are less than 18 years arrested for issues related to drug use in 2016 compared to 2011. Based on a national Lebanese report, 44% of 1307 secondary school students reported perceiving that it was easy/very easy to obtain.^[22]^ One of the reasons that explain increased BUD among young adults is explained by the increased anxiety due to political unrest and threatened future. Additional possible causes to the increased misuse rate could be related to the low perceived risk of benzodiazepine among users and prescribers, physicians over prescription, and inadequate patient-doctor communication regarding the high misuse and risks of benzodiazepines. Mitigation strategies should include a close monitor of the prescription patterns and treatment duration of benzodiazepine specifically for young adults, restricting benzodiazepine prescription to specialized physicians, physician awareness on proper patient communication regarding benzodiazepine misuse and prescription indications. Finally, awareness campaigns in schools and universities targeted at young adults are essential to increase awareness of benzodiazepine misuse and risks. A better national understanding of the national benzodiazepine uses and misuse among Lebanese young adults is required to better mitigate the associated risks and interventions.

This study has some limitations. It is a cross sectional study over a period of 10 months in 1 institution and may not represent the national data. As most ED patients are covered through private insurance, they reflect middle-to-high income class patients. The setting of the study however includes the largest ED in the country. Moreover, the study was initially conducted face-to face then shifted to a phone interview due to COVID-19 ED restriction visits. Our response rate however was high and phone interviews continued to capture all elements of the initial survey.

## Author contributions

**Conceptualization:** Tharwat El Zahran, Ziad Kazzi, Eveline Hitti.

**Data curation:** Elie Kanaan, Lynn Kobeissi, Joseph Bouassi, Aseel Sarieddine.

**Investigation:** Tharwat El Zahran.

**Methodology:** Tharwat El Zahran, Joseph Carpenter.

**Project administration:** Tharwat El Zahran, Ziad Kazzi.

**Resources:** Tharwat El Zahran.

**Software:** Tharwat El Zahran.

**Supervision:** Tharwat El Zahran, Eveline Hitti.

**Validation:** Tharwat El Zahran, Joseph Carpenter, Ziad Kazzi, Eveline Hitti.

**Visualization:** Tharwat El Zahran.

**Writing – original draft:** Elie Kanaan, Lynn Kobeissi, Joseph Bouassi, Aseel Sarieddine.

**Writing – review & editing:** Tharwat El Zahran, Joseph Carpenter, Ziad Kazzi, Eveline Hitti.

## Supplementary Material


