# Supplementary material for: Benzodiazepine use disorder: A cross-sectional study at a tertiary care center in Lebanon
Source: Medicine (Baltimore). 2022 Sep 23;101(38):e30762. doi: 10.1097/MD.0000000000030762 (PMC9509078; doi:10.1097/MD.0000000000030762)
Supplement: Supplementary file 1 [file medi-101-e30762-s001.pdf]

Appendix A: Study Questionnaire

Study ID<br>معرف الدراسة	
**Demographics**<br>**التركيبة السكانية**	
Age<br>العمر	
Gender<br>الجنس	☐ Male ذكر<br>☐ Female أنثى
Marital status<br>الوضع العائلي	☐ Married متأهل<br>☐ Widowed أرمل<br>☐ Divorced/Separated مطلق/منفصل<br>☐ Single أعزب
Smoking<br>التدخين	☐ Current smoker مدخن حالي<br>☐ Ex-smoker مدخن سابق<br>☐ None غير مدخن أبداً
Alcohol<br>الكحول	☐ Daily يومياً<br>☐ Frequently في الكثير من الأحيان<br>☐ Occasionally في القليل من الأحيان<br>☐ None لا اشرب الكحول
Other Substances<br>مواد أخرى	☐ Opioids المواد الأفيونية<br>☐ Ecstasy اكستاسي<br>☐ Marijuana الحشيشة<br>☐ Cocaine الكوكايين<br>☐ Heroin الهيرويين<br>☐ Other drugs of abuse أنواع أخرى من المخدرات
Educational level<br>المستوى التعليمي	☐ No formal education دون تعليم رسمي<br>☐ School مدرسة<br>☐ Baccalaureate مدرسة ثانوية<br>☐ Technical School مدرسة فنية<br>☐ Undergraduate جامعة<br>☐ Graduate دراسات عليا
Working Status	☐ Student تلميذ<br>☐ Employed أعمل

Institutional Review Board
American University of Beirut
09 OCT 2019
APPROVED

العمل	☐ Unemployed<br>☐ Retired	لا أعمل<br>متقاعد

**Health Status**<br>**الوضع الصحي**		
Perceived overall health over the last year<br><br>تقيمك لوضعك الصحي على مدى العام السابق	☐ Excellent<br>☐ Very good<br>☐ Good<br>☐ Fair<br>☐ Poor	ممتاز<br>جيد جداً<br>جيد<br>مقبول<br>سيئ
Presence of chronic diseases<br>الأمراض الصحية المزمنة		
Medication<br><br>الأدوية		
Guarantor<br>الجهة الضامنة		
Do you have any psychiatric diseases?<br>هل تعاني من أية أمراض نفسية؟	☐ Yes<br>☐ No	نعم<br>كلا
If you answered yes to the above question, have you seen a psychiatrist or family physician for these complaints?<br><br>، إذا أجبت بنعم على السؤال أعلاه هل رأيت طبيب نفساني أو طبيب أسرة لهذه الشكاوى؟	☐ Yes<br>☐ No	نعم<br>كلا
Do you routinely follow up with a primary care physician?<br>هل تتابع بشكل روتيني مع طبيب للرعاية الأولية؟	☐ Yes<br>☐ No	نعم<br>كلا
If yes to above, what is his/her specialty?<br><br>إذا أجبت بنعم أعلاه، ما هو إختصاص هذا الطبيب؟		

Institutional Review Board
American University of Beirut

09 OCT 2019

APPROVED

**Medication details**<br>**تفاصيل عن الأدوية**	
Over the last 6 months, have you used any of the following benzodiazepines?<br>في الستة أشهر السابقة، هل استخدمت أي من هذه البنزوديازيبينيات؟	☐ Xanax (Alprazolam) زاناكس<br>☐ Alprox (Alprazolam) البروكس<br>☐ Tranquinal (Alprazolam) ترانكوينال<br>☐ Anxyl (Bromazepam) أنكسل<br>☐ Lexotanil (Bromazepam) لكزوتانيل<br>☐ Rivotril (Clonazepam) ريفوتريل<br>☐ Valium (Diazepam) فاليوم<br>☐ Lorazepam (Lorazepam لورازيبام<br>☐ Bipax (Chlordiazepoxide) بيباكس<br>☐ Ulcedex (Chlordiazepoxide) ألسيداكس<br>☐ Librax (Chlordiazepoxide) لبراكس<br>☐ Primax (Chlordiazepoxide) بريماكس<br>☐ Psicodex (Chlordiazepoxide) سيكوديكس
How long did you take benzodiazepines for?<br>(in weeks)<br>ما المدة التي استخدمت فيها البنزوديازيبينات؟ (عدد الاسابيع)	
How often do you use benzodiazepines?<br>كم مرة تستخدم البنزوديازيبينات؟	☐ Rarely نادراً<br>☐ Occasionally في بضع الأحيان<br>☐ Frequently في الكثير من الأحيان<br>☐ Daily يومياً<br>☐ Multiple times daily عدة مرات يومياً
Why do you use this medication?<br>لماذا تستخدم هذه الأدوية؟	☐ To relax or get high للإسترخاء أو الإنتشاء<br>☐ Insomnia/Helps in sleeping الأرق/ المساعدة على النوم<br>☐ Anxiety or to relieve tension القلق أو لتخفيف التوتر<br>☐ Panic attacks نوبات الهلع<br>☐ Depression or low mood الإكتئاب أو إنخفاض الحالة المزاجية<br>☐ Epilepsy/seizures داء الصرع/ نوبات الكهرباء<br>☐ Spasticity التشنج<br>☐ Other أسباب أخرى

Institutional Review Board
American University of Beirut
09 OCT 2019
APPROVED

How did you learn about these medications?<br><br>كيف علمت بوجود هذه الأدوية؟	☐ From my physician من طبيبي<br>☐ From the pharmacist من الصيدلي<br>☐ From my friends/family من الأصدقاء/ العائلة<br>☐ On my own بنفسي<br>☐ Other بطرق أخرى
How do you obtain these medications?<br><br>كيف تحصل على هذه الأدوية؟	☐ From the pharmacist, with prescription.<br>من الصيدلي، بوصفة طبيب<br><br>☐ From the pharmacist, without prescription.<br>من الصيدلي بدون وصفة طبيب<br><br>☐ From my friends/family<br>من الأصدقاء/العائلة<br><br>☐ Samples from my physician<br>عينات من طبيبي<br><br>☐ Other (please state):<br>بطرق أخرى (الرجاء التحديد<br><br>____<br>______

**Medication details** تفاصيل عن الأدوية	
Over the last 6 months, have you used any of the following anxiolytics (non-benzodiazepine) ?<br>خلال الستة أشهر السابقة، هل استخدمت أي من هذه	☐ Flupentixol/melitracen (Deanxit) ديانكزيت<br>☐ Zolpidem (Stilnox) ستيلنوكس<br>☐ Magnesium Supplement ملحق الماغنيسيوم<br>☐ Muscerol موسيرول<br>☐ Atarax (Hydroxizine) أتاراكس<br>☐ Benadryl (Diphenhydramine) بينادريل<br>☐ Toplexil (Oxomezazine) توبليكسيل<br>☐ Rhinathiol Promethazine ريناتيول بروميثيزين

Institutional Review Board
American University of Beirut
09 OCT 2019
APPROVED

-الأدوية الغير بنزوديازيبينية؟	
How long did you take the medication for? (in weeks)<br>ما المدة التي استخدمت الأدوية فيها ؟)عدد الاسابيع(	
How often do you use the medication?<br>كم مرة تستخدم الأدوية ؟	☐ Rarely نادراً<br>☐ Occasionally في بضع الأحيان<br>☐ Frequently في الكثير من الأحيان<br>☐ Daily يومياً<br>☐ Multiple times daily عدة مرات يومياً
Why do you use this medication?<br>لماذا تستخدم هذه الأدوية؟	☐ To relax or get high للإسترخاء أو الإنتشاء<br>☐ Insomnia/Helps in sleeping الأرق/ المساعدة على النوم<br>☐ Anxiety or to relieve tension القلق أو لتخفيف التوتر<br>☐ Panic attacks نوبات الهلع<br>☐ Depression or low mood الإكتئاب أو إنخفاض الحالة المزاجية<br>☐ Epilepsy/seizures داء الصرع/ نوبات الكهرباء<br>☐ Spasticity التشنج<br>☐ Other أسباب أخرى
How did you learn about these medications?<br>كيف علمت بوجود هذه الأدوية؟	☐ From my physician من طبيبي<br>☐ From the pharmacist من الصيدلي<br>☐ From my friends/family من الأصدقاء/ العائلة<br>☐ On my own بنفسي<br>☐ Other بطرق أخرى
How do you obtain these medications?<br>كيف تحصل على هذه الأدوية؟	☐ From the pharmacist, with prescription.<br>من الصيدلي، بوصفة طبيب<br>☐ From the pharmacist, without prescription.<br>من الصيدلي بدون وصفة طبيب<br>☐ From my friends/family<br>من الأصدقاء/العائلة

Institutional Review Board
American University of Beirut
09 OCT 20[illegible]
APPROVED

	☐ Samples from my physician<br>عينات من طبيبي<br><br>☐ Other (please state):<br>بطرق أخرى (الرجاء التحديد<br>____<br>__

Perceptions about the benzodiazepine medication<br>المعرفة عن الأدوية البنزوديازيبينية					
	**Strongly disagree**<br>لا أوافق إطلاقاً	**Disagree**<br>لا أوافق	**Neutral**<br>معتدل	**Agree**<br>أوافق	**Strongly agree**<br>أوافق بشدة
I am confident that I know what my benzodiazepine medication are for<br>أن واثقّ بأني أعلم ما سبب استخدمي لأدويتي البنزوديازيبينية					
I can confidently describe how to use my prescribed benzodiazepine medication<br>يمكنني أن أصف بدقة كيف يجب أن استخدم أدويتي البنزوديازيبينية					
I can confidently describe when to use all of my prescribed benzodiazepine medication<br>يمكنني أن أصف بدقة متى يجب أن آخذ كل					

Institutional Review Board
American University of Beirut
09 OCT 2019
APPROVED

من<br>أدويتي البنزوديازيبينية					
I can name all the medications I cannot take with my benzodiazepine medication<br>يمكنني أن أسمي كل الأدوية التي لا يجب أن اتناولها مع أدويتي البنزوديازيبينية					
I can name all the foods/beverages I cannot consume with each of my prescribed medications<br>يمكنني أن أسمي كل المأكولات/ المشروبات التي لا يجب أن اتناولها مع أدويتي البنزوديازيبينية					
<u>If you had received your medication through a prescription from the doctor</u>: I am satisfied with the overall explanation I received concerning my benzodiazepine medication<br><br>***<u>إذا كنت قد حصلت على الدواء بوصفة من الطبيب</u>***<br><br>أنا راض على الشرح الذي حصلت عليه من الطبيب بالنسبة لأدويتي البنزوديازيبينية					
I understand all of the possible side					

Institutional Review Board
American University of Beirut
09 OCT 2019
APPROVED

effects of my benzodiazepine medication<br>أنا أفهم كل العوارض الجانبية التي قد تنتج عن استخدمي لهذه الأدوية البنزوديازيبينية					
I believe that there is a chance I might become addicted to the benzodiazepine medication.<br>أعتقد أنه بإمكاني أن أصبح مدمن على هذه الأدوية البنزوديازيبينية					

**Misuse**<br>سوء الإستخدام	
I have obtained this medication without prescription from a doctor<br>لقد حصلت على هذا الدواء بدون وصفة من الطبيب	☐ Yes نعم<br>☐ No كلا
I have obtained this medication through a prescription from my friend/ family member who is a doctor, but WITHOUT a medical indication<br>لقد حصلت على هذا الدواء من خلال وصفة طبية من صديقي /أحد أفراد أسرتي وهو طبيب ، ولكن دون وجود سبب طبي لإستخدام هذا الدواء	☐ Yes نعم<br>☐ No كلا
If you have obtained the medication through a prescription from your doctor for an indicated medical condition, please answer the following<br>***،إذا كنت قد حصلت على الدواء من خلال وصفة طبية من طبيبك لحالة طبية محددة الرجاء الإجابة على ما يلي***	
I have used this medication in greater amounts or doses than I was prescribed<br>لقد استخدمت هذا الدواء بكمية أو عيار أكبر من الذي وصف لي	☐ Yes نعم<br>☐ No كلا
I have used this medication more often than I was told to take<br>إن عدد المرات التي استخدم فيها هذا الدواء تفوق العدد الذي وصف لي	☐ Yes نعم<br>☐ No كلا

Institutional Review Board
American University of Beirut
09 OCT 2019
APPROVED

I have used this medication for a longer duration than I was told to take<br>لقد استخدمت هذا الدواء لمدة أطول من المدة التي وصفت لي	☐ Yes<br>☐ No	نعم<br>كلا
I have used this medication for other reasons than for what it was initially prescribed for<br>لقد استخدمت هذا الدواء لأسباب أخرى غير الأسباب التي وصفت من أجلها من قبل الطبيب	☐ Yes<br>☐ No	نعم<br>كلا

**Dependence**		**الإعتماد**
I have felt a need to increase the amount of the medication I use to get the same effect<br>أشعر بأنني اضطررت أن أزيد كمية الدواء التي اتناولها من أجل الحصول على نفس النتيجة	☐ Yes<br>☐ No	نعم<br>كلا
I feel that the same amount of medication I always take is not producing the same effect anymore<br>أشعر بأن كمية الدواء التي كنت دائماً اتناولها لا تؤدي إلى نفس المفعول الأن	☐ Yes<br>☐ No	نعم<br>كلا
I feel depressed, irritable, tired or shaky whenever I'm not using this medication.<br>أشعر بالإكتئاب أو بأني سريع الغضب أو بالتعب أو بأني متزعزع عندما لا استخدم الدواء	☐ Yes<br>☐ No	نعم<br>كلا
If yes to above, please answer:<br>I feel better once I retake the medication.<br>إذا كانت الإجابة بنعم أعلاه، الرجاء الإجابة :<br>أشعر بالتحسن عندما استخدم الدواء	☐ Yes<br>☐ No	نعم<br>كلا
I sometimes take the substance in larger amounts or over longer periods than I initially intended to<br>أحياناً آخذ الدواء بكمية أكبر أو لمدة أطول من ما كنت أقصد أو أنوي في البداية	☐ Yes<br>☐ No<br>Institutional Review Board<br>American University of Beirut<br>09 OCT 2019<br>APPROVED	نعم<br>كلا

I constantly desire or have tried unsuccessfully to cut down or control my use of these medications<br><br>دائماً ما اتمنى أو قد حولت وفشلت أن أقلل من كمية الدواء التي استخدمها	☐ Yes<br>☐ No	نعم<br>كلا
I spend a lot of time and make a lot of effort to acquire this medication, use it or recover from its effect<br><br>ابذل كثيراً من الجهد أو الوقت من أجل الحصول على هذه الأدوية أو استخدمها أو لأتعافى من تأثيرها	☐ Yes<br>☐ No	نعم<br>كلا
I have had to reduce some of my social or occupational activities because of the effects of this medication.<br><br>لقد اضطررت للحد قليلاً من انشطتي الإجتماعية أو المهنية بسبب أثار هذا الدواء	☐ Yes<br>☐ No	نعم<br>كلا
I have continued using this medication despite having a physical or psychological problem which is likely due to using it<br><br>ل لقد استمريت بإستخدام هذا الدواء على الرغم من وجود مشكلة جسدية أو نفسية لدي من الأرجح أن تكون قد نتجت عن استخدامه	☐ Yes<br>☐ No	نعم<br>كلا

Institutional Review Board
American University of Beirut

09 OCT 2019

APPROVED
